# Changes in the arabinoxylan fraction of wheat grain during alcohol production

**DOI:** 10.1016/j.foodchem.2016.10.109

**Published:** 2017-04-15

**Authors:** Ondrej Kosik, Stephen J. Powers, Afroditi Chatzifragkou, Parvathy Chandran Prabhakumari, Dimitris Charalampopoulos, Linde Hess, James Brosnan, Peter R. Shewry, Alison Lovegrove

**Affiliations:** a Department of Plant Biology and Crop Science, Rothamsted Research, Harpenden AL5 2JQ, Hertfordshire, UK; b Department of Computational and System Biology, Rothamsted Research, Harpenden AL5 2JQ, Hertfordshire, UK; c Department of Food and Nutritional Sciences, University of Reading, Whiteknights, PO Box 226, Reading RG6 6AP, UK; d The Scotch Whisky Research Institute, Research Avenue North, Riccarton, Edinburgh EH14 4AP, UK

**Keywords:** DDGS, distillers dried grains with solubles, Dietary fibre, AX, arabinoxylan, Viscosity, Solubility

## Abstract

Laboratory produced DDGS samples were compared with commercial samples from a distillery and a biofuel plant. Changes in structure, solubility and content of arabinoxylan (AX) was determined. The distillation process results in a relative increase of AX content compared to the starting material. The heating and drying processes involved in the production of DDGS lead to an increased solubility and viscosity of water-extractable AX. Production of DDGS results in structural changes to the AX. There is a decrease in 2- and 3-linked arabinose oligosaccharides, that contributes to around a 50% reduction in arabinosylation in DDGS compared with the starting grains. The current study shows that laboratory-scale DDGS provide an accurate representation of the commercial scale and that the AX composition of DDGS is consistently uniform irrespective of starting material. The uniformity of DDGS and thin stillage makes them a good potential source of AX for production of prebiotics or other novel products.

## Highlights

• AX was determined in wheat grain after commercial and laboratory alcohol production.
• Samples from laboratory scale production were similar to those from commercial processing.
• The concentration, solubility and dynamic viscosity of AX increased during processing.
• AX in DDGS was less variable in composition and properties than that in whole grain.
• DDGS has potential to provide a source of AX for novel products.

## 1 Introduction

Distillers’ Dried Grains with Solubles (DDGS) and Wet Distillers Grains (WDG) are the main co-product of distilling processes during which starch from grains (wheat, barley, maize or rye) is converted into glucose and then fermented into ethanol. Bioethanol is produced for two main purposes; traditionally as beverages or potable ethanol and more recently as a biofuel for automotive transportation (Chatzifragkou et al., 2015). The whole process is optimised to gain the highest possible yield of ethanol and the composition of DDGS varies depending on differences in the grains used, their composition, and variation in processing and production (Liu, 2011). For example, up to 85% of the thin stillage (the liquid remaining after removal of solids) is concentrated, mixed with wet solids and dried down to produce DDGS (Chatzifragkou et al., 2016). It has been reported for maize that the concentrations of components which are not fermented (such as oil, proteins, minerals and non-starch polysaccharides) are increased approximately three fold when compared to the starting feedstock and are structurally relatively unchanged (Han and Liu, 2010, Kim et al., 2008, Liu, 2011). Similar trends have been reported for wheat and mixed cereal DDGS (Nuez Ortín & Yu, 2009).

Arabinoxylan (AX) is the major fibre present in DDGS of maize and wheat, the total content being similar despite maize DDGS containing more insoluble AX than wheat (Pedersen et al., 2015). The AX content of maize DDGS, calculated as the sum of its constituent arabinose and xylose, is around four times higher than in the starting grains; in the case of soluble AX this increase is even more significant, six times higher when compared to the starting material. There is also a significant decrease in arabinose:xylose ratio when compared to the grain AX (Pedersen, Dalsgaard, Knudsen, Yu, & Lærke, 2014), indicating structural changes in the AX. Whereas the water-extractable AX comprises between 5 and 10% of total AX in maize DDGS, in wheat DDGS the portion of water-extractable AX is about 28–36% of the total (Pedersen et al., 2014, Widyaratne and Zijlstra, 2007).

These differences in AX solubility may relate to differences in their molecular structure and in their cross-linking with other components in the cell wall. The basic structure of AX in wheat and maize grains is similar, with 1,4-linked β-d-xylopyranoses making up the backbone and α-l-arabinofuranose decorations at the O-2 or/and O-3 positions of xylose units (Rose, Patterson, & Hamaker, 2010). However, more complex forms of AX occur in both species. In wheat, the AX present in the outer layers of the grain (pericarp and testa) is more highly substituted, including with glucuronic acid and *p*-coumaric acid, and is highly cross-linked (see below) resulting in low solubility. In maize bran, arabinoxylan is more complicated as the backbone can be additionally decorated with α-glucopyranosyl uronic acids and with branched side chains comprising arabinose, xylose, galactose residues and ferulic acid (Saulnier & Thibault, 1999). In both species, the O-3 linked arabinose units present as single substitutions on the xylose backbone can be esterified at the O-5 position with ferulate which can oxidatively cross-link to ferulate on an adjacent AX chain or other components to form diferulates (Huisman et al., 2000, Saulnier et al., 2007). Feruolylation is more abundant in the AX present in the outer layers (aleurone, pericarp, testa) of the grain than in the starchy endosperm. Diferulates (DFA) may play a significant role in cross-linking various cell wall components which generates insoluble complexes. It has been estimated that insoluble AX contains 8–39 times more DFA compared to soluble AX and the ratio of DFA to xylose is about five times higher in maize than in wheat insoluble fibre (Bunzel, Ralph, Marita, Hatfield, & Steinhart, 2001).

In addition to varying between grain tissues, the amount and structure of AX also varies between different wheat cultivars (Saulnier et al., 2007), and the proportion of water-extractable AX is also affected by environmental conditions during grain development (Shewry et al., 2010, Shewry et al., 2013).

The second major fibre component in cereal grains is mixed-linkage glucan (MLG) which comprises stretches of β-1,4-linked glycopyranoses interspersed with β-1,3-linkages (Buckeridge, Rayon, Urbanowicz, Tiné, & Carpita, 2004). In wheat starchy endosperm, MLG accounts for 20% of the total non-starch polysaccharides (Nemeth et al., 2010) whereas in maize the MLG content is four times lower. When the composition of the aleurone layer is taken into account, the content of MLG in wheat endosperm is ten times higher than in maize (Knudsen, 2014).

The European Food Safety Authority (EFSA) defines dietary fibre (DF) as non-digestible carbohydrates plus lignin (European Food Safety Authority, 2010). The main types of dietary fibre include resistant starch, cellulose, hemicelluloses (including xylans, pectins and β-glucans) and oligosaccharides (notably fructans). In general, these are carbohydrate polymers with 10 or more monomeric units that are not hydrolysed or absorbed in the small intestine but fermented in the colon to give short chain fatty acids (SCFAs) (Jones, 2014, Nantel, 1999). Dietary fibre has a number of health benefits (Scientific Advisory Committee on Nutrition, 2015) which are recognised by regulatory authorities in the form of accepted health claims. Furthermore, in many countries consumer intakes of fibre fall far short of recommended daily intakes, for example, approximately 18 g per day in the UK compared with a recommendation of 25–30 g (Scientific Advisory Committee on Nutrition, 2015). One strategy to exploit the health benefits of fibre in the colon is the development of prebiotics. These are types of fibre that are selectively fermented and stimulate the growth of beneficial bacteria producing SCFAs (Gibson, Probert, Van Loo, Rastall, & Roberfroid, 2004); mainly acetate, propionate and butyrate (Hopkins et al., 2003), that are considered to be responsible for a range of health benefits (Broekaert et al., 2011, Verspreet et al., 2016). We are therefore exploring the potential to use in-production DDGS fractions as a novel source of dietary fibre for human health, including the development of prebiotics, and report here the changes in AX structure which occur during DDGS production.

## 2 Materials and methods

### 2.1 Raw material

Four wheat cultivars, Claire, Istabraq, Viscount and Warrior grown at Rothamsted Research in two consecutive years (2012 and 2013) were used for laboratory scale production of Distillers’ Dried Grains with Solubles (DDGS) by The Scotch Whisky Research Institute Edinburgh, potential spirit yield was determined (Agu, Bringhurst, & Brosnan, 2006) and wet distillers grains (WDG) and thin stillage (TS) samples provided for analysis. Commercial samples, including wheat and barley grains, WDG, TS and DDGS, were kindly supplied by a UK-based potable alcohol producer (blending 95% wheat and 5% barley) and DDGS from a leading UK biofuel producer (unknown blending). Samples were freeze-dried to determine dry matter content and dried WDG and DDGS samples were ball-milled prior to analyses.

### 2.2 Alcohol yield analysis

The method used (Agu et al., 2006) simulates conditions of the production process in a ‘typical’ Scotch whisky grain distillery in respect of temperature, mashing and fermentation. Thirty g of cereal flour obtained by milling the grains in a Bühler Miag disc mill (0.2 mm setting) was transferred into a stainless steel mashing beaker and slurried with 81 ml of water and 25 μL of Termamyl 120L (industrial bacterial α-amylase; Novozymes) added. The contents of the beaker were heated to 85 °C, (2 °C/min temperature rise) in a water bath before being pressure cooked at 142 °C in an autoclave for 15 min. The cooked slurry was cooled down to 85 °C and given a second Termamyl treatment for 30 min to prevent starch retrogradation. The mash was then transferred to a 65 °C water bath and mashed for 1 h with a calculated amount of high enzyme grain distilling malt grist (Miag setting 0.2 mm), an equivalent to a malt inclusion ratio of 20% (dry weight basis) to 80% wheat. After cooling to room temperature, the mash was transferred to a fermentation vessel, pitched with distiller’s yeast (‘M’ type; Kerry Bioscience Ltd.) at a pitching rate of 0.4% (w/w) pressed yeast and adjusted to 250 g with water. Finally, the mash was fermented at 30 °C for 68 h and distilled to collect alcohol (Bringhurst & Brosnan, 2014). Alcohol yield or potential spirit yield (PSY) was determined from alcohol strength of the distillate that was measured using a Paar 500 density meter and is quoted as litres of alcohol per tonne (LA tonne^−1^) on a dry weight basis.

### 2.3 Determination of arabinoxylan content and solubility

A colorimetric method based on Finnie, Bettge, and Morris (2006) measuring pentose sugars content was used to determine total AX (TOT-AX) and water-extractable AX (WE-AX). Briefly, 125 mg of sample were suspended in 25 mL of water, vortexed and 1 ml aliquots, in duplicate, were transferred to new Pyrex tubes (fraction 1, total AX). The remainder of the suspended sample was mixed on a Spiramix tube roller for 30 min and then centrifuged at 2500×*g* for 10 min. One mL aliquots of the supernatant, in duplicate, were transferred to new Pyrex tubes (fraction 2, water-extractable AX). All fractions were diluted to 2 ml and 10 ml of freshly prepared extraction solution (93.2% (v/v) acetic acid (Sigma), 1.69% (v/v) hydrochloric acid (Fisher Scientific), 0.85% (w/v) phloroglucinol (Sigma) and 0.017% (w/v) glucose (Sigma) was added to each sample, according to the method described by Douglas (1981). Samples were boiled for 25 min and vortexed frequently. After rapid cooling the absorbance of the resulting solution was measured. The pentosan content was determined by comparing the differences in absorbance measured at 552 and 510 nm (Jenway, Bibby Scientific). Values were estimated based upon a calibration curve generated using known amounts of xylose (Sigma) standard. Water un-extractable AX (WU-AX) was calculated as the difference between TOT-AX and WE-AX and AX solubility was calculated as the ratio of WE-AX to TOT-AX.

### 2.4 Enzymatic fingerprinting of arabinoxylan and β-glucan

The protocol to analyse the AX and β-glucan structure was adapted from (Saulnier et al., 2009) using highly specific recombinant glycosylhydrolases. 100 mg of sample was suspended in 1 mL of 80% (v/v) ethanol and heated at 95 °C for 10 min to inactivate endogenous enzymes. After centrifugation at 13,400×*g* for 2 min the step was repeated with 80% (v/v) and 95% (v/v) ethanol, discarding the supernatant after each wash. The washed pellet was dried *in vacuo*. Two μL of xylanase 11 (≈11 μg, NpXyn11A, Prozomix UK) and 1 μL of lichenase (≈0.35 U, CtGH26, Prozomix UK) were added to the dried sample and made up to 1 mL with water. Samples were incubated at 40 °C in a Thermomixer (Eppendorf) with continuous shaking (750 rpm) for 16 h. Samples were then centrifuged at 13,400×*g* for 5 min, the supernatants boiled for 30 min to inactivate hydrolases and filtered through 0.45 μm PVDF filter (Whatman). Finally samples were diluted 1:20 with water prior to separation by HPAEC-PAD (Dionex ICS-3000, Thermo Scientific) equipped with a CarboPac PA1 analytical column (3 × 150 mm, Thermo Scientific) and guard (3 × 30 mm) column using the method of (Ordaz-Ortiz, Devaux, & Saulnier, 2005) modified by (Nemeth et al., 2010). Chromeleon 7 software (Thermo Scientific) was used to interpret the chromatograms. The percentage of each oligosaccharide originating from the fingerprinted AX (for nomenclature used see Fauré et al., 2009), the percentages of xylose, 3- and 2-linked arabinose, as well as total arabinosylation and the arabinose:xylose ratio were calculated. The% arabinosylation was calculated using the full fingerprint data (Table S1). The peak areas of those oligosaccharides with arabinose additions was divided by the total peak area for all of AXOS peaks generated by the enzyme digests and quoted as percent. The G3:G4 ratio (Toole et al., 2010) reflecting the changes in β-1,3- and β-1,4-linkages in mixed-linkage glucan, was also calculated.

### 2.5 Dynamic viscosity

The dynamic viscosity of aqueous extracts prepared according to the protocol of (Saulnier, Peneau, & Thibault, 1995) were measured using a Micro-Ostwald capillary viscometer (AVS370, SI Analytics Germany) at 30 °C (Freeman et al., 2016). Briefly, 1 g of sample was suspended in 4 ml of water, vortexed and mixed on a Spiramix tube roller for 15 min. After centrifugation at 5000×*g* for 10 min the supernatant was collected and boiled for 10 min. Precipitated proteins were removed by centrifugation at 5000×*g* for 10 min. The supernatant was centrifuged again (10,000*g* for 10 min) filtered through a 0.45 μm PVDF filter and the dynamic viscosity measured.

### 2.6 Statistical analyses

The method of principal coordinate analysis (PCoA) was used to give an overall low-dimensional description of the data. This method analyses the matrix of similarities between *n* samples calculated using a simple Euclidean distance measure in multivariate space between the samples. A number of principal coordinates (PCos) are retained that account for the majority of the variance in the distances, thus allowing visualisation of the differences between samples in 2-d plots (Krzanowski, 2000). Afterwards, relating the retained principal coordinates (PCos) to the original variables via correlation analysis enabled us to see the variables most correlated with the separation in each dimension. This was done by obtaining the F-statistics for each correlation.

Correlations between response variables were also calculated, and tested using the F-test. All analyses were done using the GenStat (17th edition, VSN International Ltd, Hemel Hempstead, UK) statistical package. SIMCA 14 (MKS Data Analytics Solutions, Sweden) was used to generate the plots.

## 3 Results and discussion

### 3.1 Potential spirit yield and variation of TOT-AX in grains

Four wheat cultivars grown at Rothamsted Research in 2012 and 2013 and were used to determine changes in AX amount and composition during alcohol production. Three of these cultivars are or were commonly used for distilling (Claire, Istabraq and Viscount) and were expected to give high yields of alcohol. The fourth cultivar, Warrior, is known to give low alcohol yields and is therefore not usually used for distilling. Commercial samples, including wheat and barley grains, WDG, TS and DDGS from a UK-based potable alcohol producer and DDGS from a leading UK biofuel producer, all from 2012, were also analysed. Samples of the grains from the commercial biofuel plant were not available for comparison.

The results are summarised in Table 1, including total (TOT-AX), water-extractable (WE-) AX and AX solubility, calculated as the percentage of water-extractable AX. The dynamic viscosities of aqueous extracts were also measured and the potential spirit yields were determined. The mean total AX content of these samples was 1.5-fold higher than in the grain used in the commercial distillery (which comprised 95% wheat and 5% barley grains). As expected, Warrior gave the lowest spirit yield in 2012, but this was slightly higher than for Claire in 2013 (both cultivars being substantially below Istabraq and Viscount)

### 3.2 Compositional analysis of fractions from alcohol production

**Fig. 1:**
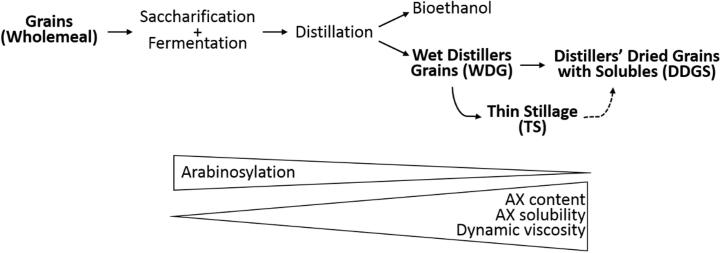
Flowchart showing the production of bioethanol and DDGS and the effects on arabinoxylan (AX) content and structure.

The production of alcohol from wheat grain (Fig. 1) results in two “waste streams”, corresponding to soluble components (thin stillage, TS) and the insoluble grain residue (wet distillers’ grains, WDG). In commercial distilleries and biofuel plants a proportion of the TS is sprayed onto the WDG and dried to give distillers’ dried grains with solubles (DDGS). For the laboratory scale preparations individual fractions were retained and the WDG alone dried to give DDGS, as it was not known what proportion of TS is sprayed onto WDG in the commercial distillery and biofuel plant. All fractions from the laboratory scale and commercial preparations were analysed for TOT-AX and WE-AX and the dynamic viscosity of aqueous extracts measured and AX solubility calculated. The results are included in Table 1.

### 3.3 Increase in AX content and AX solubility

All three processed fractions (TS, WDG, DDGS) are richer in TOT-AX than the starting material which is to be expected as starch accounts for about 70% of the mature dry grain and would be removed by hydrolysis and fermentation. The increase in total AX in the DDGS from the commercial distillery was 4.5-fold, (Table 1) compared with the raw material (95% wheat, 5% barley). A similar increase was previously reported for commercial maize DDGS (3.9-fold; Pedersen et al., 2014). The increase in TOT-AX was less for the four laboratory samples, about 1.8 to 2.8-fold.

The DDGS fractions also had somewhat higher contents of WE-AX than the starting material. There was an 11-fold increase in water-extractable AX (WE-AX) for the commercial distilling samples and the WE-AX content of the DDGS from the biofuel production was also high (although in this case a comparison with the starting grains could not be made). The DDGS produced in the laboratory had between 3.2 and 3.7-fold more WE-AX than the starting grain. A 2.7-fold increase in WE-AX content was previously reported for wheat DDGS (Widyaratne & Zijlstra, 2007) from a Canadian ethanol plant.

The differences in the degree of enrichment of TOT-AX and WE-AX between the commercial and laboratory scale DDGS samples probably results from the fact that the TS was sprayed back onto the DDGS in the commercial samples but not those prepared in the laboratory (Chatzifragkou et al., 2015).

When expressed as a percentage of TOT-AX, the proportion of WE-AX increased from 22.8% in flour to 56% in the distillery DDGS and was 38.2% in the biofuel sample (where no starting material was available). For the laboratory scale samples, WE-AX increased on average (over cultivars) from 19.9% for wholemeal flour to 30.7% for DDGS in 2012 and from 22.8% to 29.9% in 2013.

### 3.4 Increase in dynamic viscosity

The values for dynamic viscosity of extracts of the laboratory scale and distillery samples of WM, TS and WDG were very similar, as were those of the DDGS samples from the distillery and biofuel plants (summarised in Table 1). The differences between the dynamic viscosities of extracts of the commercial and laboratory scale samples of DDGS probably relate to the absence of the TS from the latter.

### 3.5 Changes in AX and glucan structure

The structures of AX and MLG in the samples were determined by enzymatic fingerprinting and HPAEC-PAD. The oligosaccharides released by enzymatic digest had previously been identified (Anders et al., 2012, Ordaz-Ortiz et al., 2005) allowing the proportions of xylose and 3- and 2-linked arabinose, the total arabinosylation, the arabinose:xylose ratio, the total AX peak area and the proportions of the individual fragments to be compared. The total MLG peak area (the sum of G3 and G4 peak areas), the G3:G4 ratio and the total AX and glucan peak areas were also calculated. The data discussed below are summarised in Table 2 while complete datasets are provided in Supplementary Table S1.

Arabinosylation decreased during processing, from means of 12.1% (2012) and 13.7% (2013) over cultivars for the raw material to 6.4% (2012) and 6.8 (2013) for the WDG and to 6.2% (2012) and 6.8% (2013) for DDGS. Similar values of percentage arabinosylation were obtained for the commercial distillery, biofuel samples and laboratory-scale samples, indicating that the laboratory-scale samples replicate the commercial process. The TS samples had similar% arabinosylation to the starting material. Although this may indicate that they have similar structures, all of the AX in the TS was water-extractable, whereas only between 17% and 26% of the total AX is water-extractable in the raw material (Table 1). This demonstrates that the solubility of AX in wheat is not determined by the degree of modification with arabinose. By contrast, the percentage of arabinosylation of the WDG and DDGS fractions was lower by about half, than that of the starting material or TS, indicting a change in structure. The enzymatic fingerprinting also demonstrated that these fractions had increased proportions of xylose and xylobiose (X_2_) fragments, which indicates that arabinose is lost during processing. This is consistent with the digestion pattern of oligosaccharides observed using the recombinant xylanase enzyme from the GH11 family, which hydrolyses only at unsubstituted xylan residues (Kolenová, Vršanská, & Biely, 2006; Biely, Vrsanska, Tenkanan, & Kluepfel, 1997).

### 3.6 Multivariate analyses

**Fig. 2:**
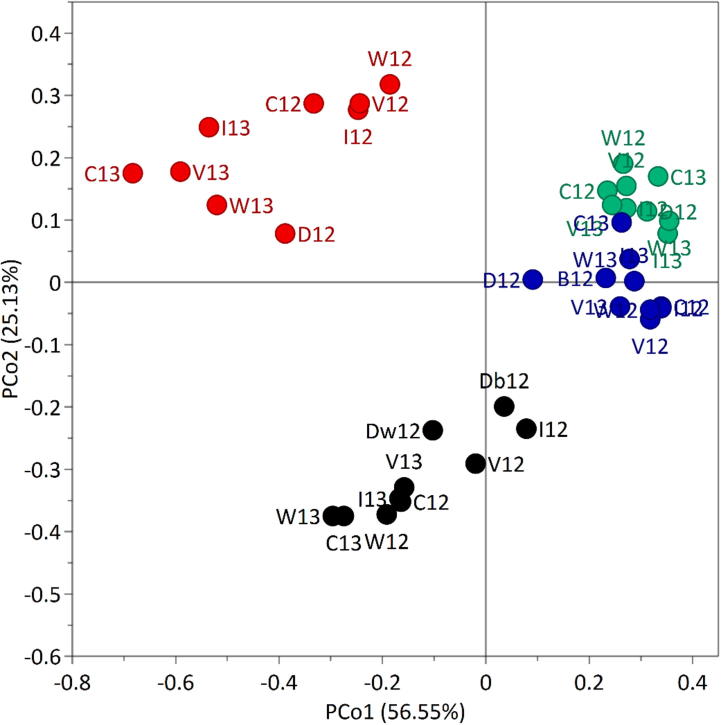
Principal coordinates analysis (PCoA) plot for analyses of compositional data summarised in Table 1, Table 2 using the following coding of samples for cultivars: Claire (C), Istabraq (I), Viscount (V), Warrior (W), commercial distillery wheat (Dw) and barley (Db), and biofuel (B); followed by year: 2012 (12) and 2013 (13) and coloured by fraction type: wholemeal (WM, coloured black), thin stillage (TS, coloured red), wet distillers grains (WDG, coloured green) and distillers’ dried grains with solubles (DDGS, coloured blue). The percentage of the variation in the distances between the samples accounted for by each PCo is shown in brackets. The first two PCos accounted for 81.68% of the variation in the distances between the samples and so only these two are retained for visualisation of the samples. (For interpretation of the references to color in this figure legend, the reader is referred to the web version of this article.)

In order to visualise the similarities or differences between the samples and to identify which underlying variables may be responsible for the separation of them, Principal Coordinates Analysis (PCoA) was applied to the compositional dataset in Table 1, the fingerprinting data in Table 2 and the combined datasets. Although these three analyses showed broadly similar separations, the degree of discrimination is greater when the full dataset was used. This is shown in Fig. 2 with the analyses of the data in Table 1, Table 2 being shown in Supplementary Fig. S1. The PCoA plot in Fig. 2 shows wholemeal flour (WM, coloured black) and thin stillage (TS, coloured red) being separated from wet distillers grains (WDG, coloured green) and distillers’ dried grains with solubles (DDGS, coloured blue) on the PCo1 axis, and TS and WDG being separated from WM and DDGS on the PCo2 axis with the exception of Claire 2013 DDGS. The grouping of the fractions from commercial and laboratory scale production together confirms that the laboratory scale system is a good model for commercial distilling and bioethanol production.

The F-statistics for correlations of variables with the PCo1 and PCo2 values from Fig. 2 were calculated in order to identify the most discriminatory variables. In the PCo1 direction the most discriminatory, top five variables are the percentage of 2-linked arabinose (F-PCo1 = 717.7), followed by xylobiose oligosaccharide (685.6) and XA^2+3^XX oligosaccharide as a percentage of total fingerprinted AX peak area (680.1), the percentage of unsubstituted xylose and percentage of arabinosylation (both 655.4) and finally the arabinose:xylose ratio (644.0). These variables are probably responsible for the separation of the WM and TS from the WDG. In the PCo2 direction the most discriminatory variables were the ‘AX + MLG total peak area’ (F-PCo2 = 136.0) and ‘AX total peak area’ (117.2). These variables are responsible for discriminating the TS and WDG from the WM and DDGS fractions (except for Claire 2013 DDGS), and also for the separation of the distillery TS from other TS fractions.

The PCoA also shows differences between the laboratory samples from the two growth years, particularly in the compositions of the TS (coloured red) and WM (coloured black) samples (Fig. 2). The distillery WM and TS samples also differ from the laboratory scale samples whereas the WDG sample clusters well with the laboratory scale samples from both years. The separation of the distillery DDGS from the other DDGS samples can be explained by slightly higher arabinose content that probably results from the TS (which contains WE-AX) being back sprayed onto the DDGS during processing. The separation of the 2013 Claire sample of DDGS from the other DDGS samples, on PCo2, is primarily due to the higher MLG content, shown as a greater MLG peak area in Table 2.

### 3.7 Associations between solubility, viscosity and AX structure changes

**Fig. 3:**
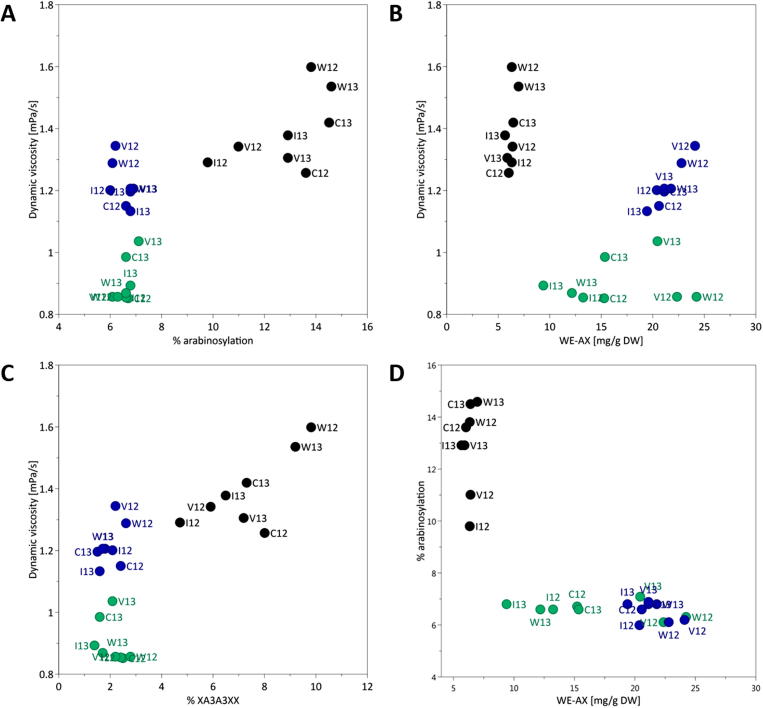
Relationships between the AX structure, AX solubility of and dynamic viscosity of laboratory scale samples of wholemeal (WM, coloured black), wet distillers grains (WDG, coloured green) and DDGS (D, coloured blue). (A) dynamic viscosity *vs.* percentage arabinosylation; (B) dynamic viscosity *vs.* amount of WE-AX; (C) dynamic viscosity *vs.* percentage of XA3A3XX; (D) percentage of arabinosylation *vs.* amount of WE-AX. (For interpretation of the references to color in this figure legend, the reader is referred to the web version of this article.)

The major changes observed during DDGS production were increased AX content and solubility, dynamic viscosity and decreased arabinosylation, as summarised in Fig. 1. These relationships are explored in more detail in Fig. 3 for the WM, WDG and DDGS samples from the four wheat cultivars; a shade plot of the matrix of correlations using all laboratory scale and commercial samples is given in Supplementary Fig. S2. Although dynamic viscosity was significantly correlated with the percentage of arabinosylation calculated from the enzyme mapping, (*r* = 0.722, *p* < 0.001, *n* = 24) overall, there were no significant (p < 0.05) correlations within the three types of grain sample: unprocessed wholemeal, WDG and DDGS (Fig.3A). Whereas the wholegrain samples varied in both percentage arabinosylation and dynamic viscosity these were not related, while the WDG and DDGS samples each formed a tight group. These samples had similar low levels of arabinosylation but the DDGS samples had higher dynamic viscosity. A similar pattern was observed when dynamic viscosity was plotted against the percentage of the XA^3^A^3^XX arabinoxylan oligosaccharide (AXOS) released by endoxylanase digestion (*r* = 0.730, *p* < 0.001, *n* = 24) (Fig.3C). This AXOS made a relatively minor contribution to the total AXOS released from wholegrain (between 1.6 and 2.3%) but showed the greatest proportional decrease during processing, to 0.6–0.7% in the DDGS.

Broadly similar plots were observed when WE-AX was plotted against dynamic viscosity (*r* = 0.131, *p* = 0.541, *n* = 24) (Fig.3B) and percentage arabinosylation (*r* = -0.292, *p* = 0.167, *n* = 24) (Fig.3D). Although the wholegrain samples ranged widely in both dynamic viscosity and percentage arabinosylation these were not correlated with WE-AX. The WDG samples varied widely in WE-AX but not in dynamic viscosity or percentage arabinosylation, while the DDGS samples formed tighter groups with WE-AX showing a linear correlation with dynamic viscosity. The latter was the only strong correlation (*r* = 0.763, *p* = 0.028, *n* = 8) observed between the parameters plotted for the three types of sample.

These results indicate that the degree of arabinosylation is not a major factor determining the extent of AX solubility and dynamic viscosity.

However, they also demonstrate two features which are of relevance to the utilisation of DDGS for high value products. These are that the AX present in DDGS is more uniform in composition and properties than that in WDG or the starting material, and that there is no clear relationship between the compositions of the starting material and DDGS. Hence, the selection of starting material is of little importance when producing DDGS for exploitation compared to the selection of the processing parameters.

## 4 Conclusions

DDGS samples were prepared using a laboratory process and compared with samples from a commercial distillery and biofuel plant. The amount, solubility, degree of arabinosylation and structure of the major fibre components, AX and MLG, were determined. The hydrolysis and fermentation of starch from laboratory and commercial samples resulted in an increased concentration of AX, but its structure was also modified. In particular, the process of DDGS production resulted in a higher proportion of soluble AX, contributing to increased dynamic viscosity of aqueous extracts than was found in the starting material. This study shows that laboratory scale production of DDGS can be used to predict the behaviour of grain in commercial distillery and biofuel processes. Furthermore, it also demonstrates that DDGS samples are more uniform in AX composition and properties than wholemeal or WDG samples, with little or no effect of variation in composition of the wholemeal on the final co-product, and that thin stillage and DDGS are good potential sources of AX for exploitation to produce novel food ingredients and prebiotics.

## Conflict of interest statement

The authors of this paper have no affiliations with or involvement in any organization or entity with any financial or non-financial interest in the subject matter or materials discussed in this manuscript.

## Figures and Tables

**Table 1:** Analysis of grain fractions from laboratory scale alcohol production (wheat cultivars Claire, Istabraq, Viscount and Warrior grown in 2012 and 2013) and a commercial distillery and biofuel plant.

Fraction	Cultivar	Year	Total-AX [mg g^−1^ DW]	Water-extractable AX [mg g^−1^ DW]	AX solubility [%]	Dynamic viscosity [mPa s^−1^]	Potential spirit yield [LA tonne^−1^]
Wholemeal (WM)	Claire	2012	32.27	6.02	18.7	1.256	444
		2013	32.45	6.44	19.8	1.420	417
	Istabraq	2012	36.87	6.33	17.2	1.291	437
		2013	25.66	5.66	22.1	1.378	436
	Viscount	2012	30.09	6.42	21.4	1.341	449
		2013	25.15	5.89	23.4	1.306	449
	Warrior	2012	28.71	6.35	22.1	1.598	419
		2013	26.62	6.96	26.1	1.535	420
	Distillery	2012	19.19	4.37	22.8	1.401	–

Thin stillage (TS)	Claire	2012	64.10	64.10	100	1.290	–
		2013	63.15	63.15	100	1.380	–
	Istabraq	2012	62.85	62.85	100	1.310	–
		2013	57.13	57.13	100	1.330	–
	Viscount	2012	67.24	67.24	100	1.290	–
		2013	48.96	48.96	100	1.340	–
	Warrior	2012	72.38	72.38	100	1.330	–
		2013	42.31	42.31	100	1.420	–
	Distillery	2012	58.61	58.61	100	1.323	–

Wet distillers grains (WDG)	Claire	2012	55.16	15.25	27.6	0.853	–
		2013	100.38	15.35	15.3	0.986	–
	Istabraq	2012	61.64	13.26	21.5	0.854	–
		2013	98.44	9.36	9.5	0.894	–
	Viscount	2012	68.82	22.35	32.5	0.856	–
		2013	93.45	20.45	21.9	1.037	–
	Warrior	2012	75.77	24.24	32.0	0.857	–
		2013	110.14	12.18	11.1	0.868	–
	Distillery	2012	75.88	7.82	10.3	0.861	–

Distillers’ dried grains with solubles (DDGS)	Claire	2012	91.43	20.57	22.5	1.151	–
		2013	68.67	21.10	30.7	1.195	–
	Istabraq	2012	66.02	20.35	30.8	1.200	–
		2013	71.52	19.40	27.1	1.134	–
	Viscount	2012	72.82	24.11	33.1	1.343	–
		2013	64.73	21.13	32.6	1.207	–
	Warrior	2012	62.67	22.80	36.4	1.289	–
		2013	75.24	21.80	29.0	1.205	–
	Distillery	2012	85.78	48.07	56.0	2.316	–
	Biofuel	2012	90.22	34.50	38.2	2.304	–

The wholemeal samples are the raw material which for the distillery comprised 95% wheat and 5% barley (cultivars not known). Only DDGS was available from the biofuel plant. Arabinoxylan (AX) solubility is expressed as a percentage of WE-AX. The potential alcohol yield from the laboratory scale samples is also given. It is notable that the alcohol yield for Warrior was lower than for the other cultivars grown 2012, but similar to that of Claire (both being low) in the samples grown in 2013.

**Table 2:** Analysis of the structures of arabinoxylan (AX) and mixed-linkage glucan (MLG) by enzyme fingerprinting. For nomenclature see (Fauré et al., 2009). Arabinosylation data is also included, along with AX + MLG and MLG peak area from HPAEC-PAD. Only data which are discussed in the text are included, with full datasets being provided as Supplementary data (Table S1).

Fraction	Cultivar	Year/grain	Xyl + Xyl_2_[%]	XA^3^XX[%]	XA^2+2^XX[%]	XA^3^XA^3^XX[%]	XA^3^A^2+2^XX[%]	Arabinosylation[%]	AX + MLGPeak area	MLGPeak area
Wholemeal (WM)	Claire	2012	49.5	21.2	13.7	1.6	2.9	13.6	169.96	37.28
		2013	48.6	16.6	15.9	2.3	3.8	14.5	111.08	27.13
	Istabraq	2012	63.0	17.1	9.5	1.2	2.0	9.8	222.25	52.07
		2013	53.1	17.9	13.1	1.9	2.9	12.9	108.09	33.12
	Viscount	2012	59.3	16.7	11.7	1.5	2.5	11.0	164.17	34.95
		2013	54.2	15.5	14.2	1.6	3.6	12.9	131.46	34.88
	Warrior	2012	48.1	22.3	12.3	1.8	2.7	13.8	119.62	28.54
		2013	48.5	15.9	14.9	2.1	3.8	14.6	120.92	35.47
	Distillery	Wheat	56.2	18.1	12.5	1.4	2.5	11.9	137.55	22.64
		Barley	63.4	18.3	8.6	2.6	2.6	9.5	111.16	21.70

Thin stillage (TS)	Claire	2012	54.9	16.3	14.3	2.6	2.6	12.4	419.36	45.27
		2013	38.5	18.2	21.5	2.7	5.7	17.7	429.90	116.15
	Istabraq	2012	59.9	15.5	9.7	2.6	2.9	10.9	359.20	41.97
		2013	45.2	19.9	17.4	2.7	4.0	15.3	513.75	188.57
	Viscount	2012	61.3	13.6	11.1	2.1	3.0	10.7	360.88	110.90
		2013	43.9	17.5	18.6	2.5	5.3	16.0	437.84	169.48
	Warrior	2012	62.5	15.0	9.5	1.9	2.3	10.1	368.32	94.61
		2013	46.5	18.3	16.6	2.4	4.6	15.1	329.17	124.81
	Distillery	2012	50.2	16.1	14.9	1.8	3.6	13.9	243.62	49.55

Wet distillers grains (WDG)	Claire	2012	73.3	15.2	5.7	0.8	1.3	6.7	335.01	56.70
		2013	72.7	18.3	5.0	0.5	1.1	6.6	393.98	69.49
	Istabraq	2012	73.2	16.5	4.9	0.8	1.1	6.6	291.36	58.11
		2013	71.2	20.4	4.6	0.5	1.1	6.8	285.83	60.88
	Viscount	2012	75.7	14.0	5.3	0.7	1.1	6.1	280.52	49.15
		2013	71.9	16.2	6.4	0.6	1.6	7.1	294.22	66.22
	Warrior	2012	75.0	14.1	5.1	0.8	1.1	6.3	319.73	54.96
		2013	72.5	18.5	4.8	0.5	1.2	6.6	290.04	58.98
	Distillery	2012	72.5	16.7	5.3	0.7	1.2	6.8	381.93	66.62

Distillers’ dried grains with solubles (DDGS)	Claire	2012	73.8	15.5	5.4	0.7	1.1	6.6	312.26	52.26
		2013	72.6	16.9	6.0	0.6	1.2	6.8	428.53	89.93
	Istabraq	2012	75.4	15.4	4.6	0.7	0.9	6.0	295.94	57.69
		2013	72.7	16.9	5.7	0.7	1.2	6.8	330.11	74.70
	Viscount	2012	75.5	14.3	5.3	0.7	1.1	6.2	256.24	46.63
		2013	73.2	14.9	6.5	0.7	1.5	6.9	347.48	74.57
	Warrior	2012	75.4	14.5	4.9	0.6	1.0	6.1	284.28	50.25
		2013	72.8	16.2	5.9	0.7	1.3	6.8	373.90	77.96
	Distillery	2012	67.1	15.2	8.1	1.1	2.0	8.7	349.59	48.51
	Biofuel	2012	72.1	14.2	6.2	1.0	1.6	7.2	362.22	35.30
