# Supplementary material for: Changes in the arabinoxylan fraction of wheat grain during alcohol production
Source: Food Chem. 2017 Apr 15;221:1754–62. doi: 10.1016/j.foodchem.2016.10.109 (PMC5176037; doi:10.1016/j.foodchem.2016.10.109)
Supplement: Supplementary data 1 [file mmc1.docx]

**Table S1.** The complete dataset used in the paper. Additionally, water un-extractable AX, 3-Ara : 2-Ara ratio, Ara : Xyl ratio and Total AX peak area were calculated using data presented in this table.

| Fraction | Cultivar | Year | Dry matter  [%] | Total-AX  [mg.g^-1^ DW] | Water-extractable AX  [mg.g^-1^ DW] | AX solubility  [%] | Dynamic  viscosity  [mPa.s^-1^] | Xyl [%] | Xyl_2_  [%] | XA^3^XX  [%] | XA^2+2^XX  [%] | XA^3^XA^3^XX  [%] | XA^3^A^2+2^XX  [%] | Arabino-  sylation  [%] | Xyl | 3-Ara | 2-Ara | 3-Ara:2-Ara | AX+MLG  Peak area | MLG  Peak area | G3:G4 |
| --- | --- | --- | --- | --- | --- | --- | --- | --- | --- | --- | --- | --- | --- | --- | --- | --- | --- | --- | --- | --- | --- |
| Wholemeal  (WM) | Claire | 2012 | 92.30 | 32.27 | 6.02 | 18.7 | 1.256 | 15.4 | 34.1 | 21.2 | 13.7 | 1.6 | 2.9 | 13.6 | 86.4 | 10.6 | 3.0 | 3.536 | 169.96 | 37.28 | 2.72 |
|  |  | 2013 | 88.87 | 32.45 | 6.44 | 19.8 | 1.420 | 17.9 | 30.7 | 16.6 | 15.9 | 2.3 | 3.8 | 14.5 | 85.5 | 10.8 | 3.7 | 2.891 | 111.08 | 27.13 | 2.01 |
|  | Istabraq | 2012 | 92.24 | 36.87 | 6.33 | 17.2 | 1.291 | 22.1 | 40.9 | 17.1 | 9.5 | 1.2 | 2.0 | 9.8 | 90.2 | 7.7 | 2.1 | 3.672 | 222.25 | 52.07 | 2.79 |
|  |  | 2013 | 88.63 | 25.66 | 5.66 | 22.1 | 1.378 | 18.3 | 34.8 | 17.9 | 13.1 | 1.9 | 2.9 | 12.9 | 87.1 | 9.8 | 3.1 | 3.214 | 108.09 | 33.12 | 2.09 |
|  | Viscount | 2012 | 88.09 | 30.09 | 6.42 | 21.4 | 1.341 | 18.8 | 40.5 | 16.7 | 11.7 | 1.5 | 2.5 | 11.0 | 89.0 | 8.5 | 2.5 | 3.350 | 164.17 | 34.95 | 3.52 |
|  |  | 2013 | 88.74 | 25.15 | 5.89 | 23.4 | 1.306 | 18.6 | 35.6 | 15.5 | 14.2 | 1.6 | 3.6 | 12.9 | 87.1 | 9.7 | 3.2 | 3.002 | 131.46 | 34.88 | 2.43 |
|  | Warrior | 2012 | 88.46 | 28.71 | 6.35 | 22.1 | 1.598 | 14.9 | 33.2 | 22.3 | 12.3 | 1.8 | 2.7 | 13.8 | 86.2 | 11.1 | 2.7 | 4.036 | 119.62 | 28.54 | 2.41 |
|  |  | 2013 | 89.00 | 26.62 | 6.96 | 26.1 | 1.535 | 16.3 | 32.2 | 15.9 | 14.9 | 2.1 | 3.8 | 14.6 | 85.4 | 11.0 | 3.6 | 3.080 | 120.92 | 35.47 | 2.22 |
|  | Distillery | 2012 | 1.40 | 19.19 | 4.37 | 22.8 | 1.401 | 25.3 | 29.6 | 18.1 | 12.5 | 1.4 | 2.5 | 11.9 | 87.6 | 9.4 | 3.0 | 3.136 | 137.55 | 22.64 | 1.79 |
| Thin stillage  (TS) | Claire | 2012 | 2.56 | 64.10 | 64.10 | 100 | 1.290 | 18.7 | 19.8 | 18.3 | 8.6 | 2.6 | 2.6 | 9.5 | 82.3 | 12.8 | 4.9 | 2.623 | 111.16 | 21.70 | 1.98 |
|  |  | 2013 | 2.30 | 63.15 | 63.15 | 100 | 1.380 | 25.9 | 34.0 | 16.3 | 14.3 | 2.6 | 2.6 | 12.4 | 89.1 | 8.6 | 2.3 | 3.832 | 419.36 | 45.27 | 2.02 |
|  | Istabraq | 2012 | 2.30 | 62.85 | 62.85 | 100 | 1.310 | 18.5 | 26.7 | 18.2 | 21.5 | 2.7 | 5.7 | 17.7 | 84.7 | 11.4 | 3.9 | 2.960 | 429.90 | 116.15 | 1.94 |
|  |  | 2013 | 1.90 | 57.13 | 57.13 | 100 | 1.330 | 28.3 | 33.0 | 15.5 | 9.7 | 2.6 | 2.9 | 10.9 | 89.3 | 8.2 | 2.5 | 3.223 | 359.20 | 41.97 | 2.90 |
|  | Viscount | 2012 | 2.42 | 67.24 | 67.24 | 100 | 1.290 | 17.4 | 26.5 | 19.9 | 17.4 | 2.7 | 4.0 | 15.3 | 84.0 | 11.7 | 4.3 | 2.719 | 513.75 | 188.57 | 2.02 |
|  |  | 2013 | 1.70 | 48.96 | 48.96 | 100 | 1.340 | 28.1 | 34.4 | 13.6 | 11.1 | 2.1 | 3.0 | 10.7 | 89.9 | 8.0 | 2.1 | 3.746 | 360.88 | 110.90 | 2.48 |
|  | Warrior | 2012 | 2.45 | 72.38 | 72.38 | 100 | 1.330 | 18.8 | 27.7 | 17.5 | 18.6 | 2.5 | 5.3 | 16.0 | 84.9 | 11.2 | 3.9 | 2.902 | 437.84 | 169.48 | 2.10 |
|  |  | 2013 | 13.70 | 42.31 | 42.31 | 100 | 1.420 | 29.4 | 43.9 | 15.0 | 9.5 | 1.9 | 2.3 | 10.1 | 93.3 | 5.5 | 1.2 | 4.394 | 368.32 | 94.61 | 2.47 |
|  | Distillery | 2012 | 22.55 | 58.61 | 58.61 | 100 | 1.323 | 27.6 | 45.1 | 18.3 | 16.6 | 2.4 | 4.6 | 15.1 | 93.4 | 5.5 | 1.1 | 5.179 | 329.17 | 124.81 | 2.16 |
| Wet distillers grains (WDG) | Claire | 2012 | 13.40 | 55.16 | 15.25 | 27.6 | 0.853 | 27.7 | 45.5 | 16.1 | 14.9 | 1.8 | 3.6 | 13.9 | 93.4 | 5.5 | 1.1 | 5.175 | 243.62 | 49.55 | 2.35 |
|  |  | 2013 | 26.83 | 100.38 | 15.35 | 15.3 | 0.986 | 24.8 | 46.4 | 15.2 | 5.7 | 0.8 | 1.3 | 6.7 | 93.2 | 5.8 | 1.0 | 5.852 | 335.01 | 56.70 | 2.18 |
|  | Istabraq | 2012 | 14.00 | 61.64 | 13.26 | 21.5 | 0.854 | 28.9 | 46.8 | 18.3 | 5.0 | 0.5 | 1.1 | 6.6 | 93.9 | 5.0 | 1.1 | 4.416 | 393.98 | 69.49 | 2.60 |
|  |  | 2013 | 16.08 | 98.44 | 9.36 | 9.5 | 0.894 | 25.5 | 46.4 | 16.5 | 4.9 | 0.8 | 1.1 | 6.6 | 92.9 | 5.7 | 1.4 | 4.047 | 291.36 | 58.11 | 2.24 |
|  | Viscount | 2012 | 11.70 | 68.82 | 22.35 | 32.5 | 0.856 | 30.7 | 44.3 | 20.4 | 4.6 | 0.5 | 1.1 | 6.8 | 93.7 | 5.2 | 1.1 | 4.674 | 285.83 | 60.88 | 2.43 |
|  |  | 2013 | 24.92 | 93.45 | 20.45 | 21.9 | 1.037 | 23.4 | 49.1 | 14.0 | 5.3 | 0.7 | 1.1 | 6.1 | 93.4 | 5.6 | 1.0 | 5.406 | 280.52 | 49.15 | 2.15 |
|  | Warrior | 2012 | 96.60 | 75.77 | 24.24 | 32.0 | 0.857 | 29.4 | 44.4 | 16.2 | 6.4 | 0.6 | 1.6 | 7.1 | 93.4 | 5.4 | 1.2 | 4.612 | 294.22 | 66.22 | 2.13 |
|  |  | 2013 | 62.44 | 110.14 | 12.18 | 11.1 | 0.868 | 28.9 | 43.7 | 14.1 | 5.1 | 0.8 | 1.1 | 6.3 | 93.2 | 5.5 | 1.3 | 4.324 | 319.73 | 54.96 | 2.16 |
|  | Distillery | 2012 | 96.60 | 75.88 | 7.82 | 10.3 | 0.861 | 29.9 | 45.5 | 18.5 | 4.8 | 0.5 | 1.2 | 6.6 | 94.0 | 5.0 | 1.0 | 5.159 | 290.04 | 58.98 | 2.30 |
| Distillers’ dried  grains with  solubles  (DDGS) | Claire | 2012 | 78.15 | 91.43 | 20.57 | 22.5 | 1.151 | 28.8 | 43.9 | 16.7 | 5.3 | 0.7 | 1.2 | 6.8 | 93.2 | 5.5 | 1.2 | 4.474 | 381.93 | 66.62 | 2.21 |
|  |  | 2013 | 96.60 | 68.67 | 21.10 | 30.7 | 1.195 | 28.1 | 47.4 | 15.5 | 5.4 | 0.7 | 1.1 | 6.6 | 93.8 | 5.0 | 1.1 | 4.499 | 312.26 | 52.26 | 2.36 |
|  | Istabraq | 2012 | 98.78 | 66.02 | 20.35 | 30.8 | 1.200 | 29.0 | 44.2 | 16.9 | 6.0 | 0.6 | 1.2 | 6.8 | 93.1 | 5.4 | 1.4 | 3.816 | 428.53 | 89.93 | 2.22 |
|  |  | 2013 | 96.60 | 71.52 | 19.40 | 27.1 | 1.134 | 29.1 | 46.3 | 15.4 | 4.6 | 0.7 | 0.9 | 6.0 | 93.9 | 5.1 | 1.0 | 4.837 | 295.94 | 57.69 | 2.32 |
|  | Viscount | 2012 | 77.63 | 72.82 | 24.11 | 33.1 | 1.343 | 30.4 | 42.4 | 16.9 | 5.7 | 0.7 | 1.2 | 6.8 | 93.2 | 5.5 | 1.3 | 4.255 | 330.11 | 74.70 | 2.14 |
|  |  | 2013 |  | 64.73 | 21.13 | 32.6 | 1.207 | 21.0 | 35.2 | 14.3 | 5.3 | 0.7 | 1.1 | 6.2 | 88.1 | 9.2 | 2.7 | 3.441 | 256.24 | 46.63 | 2.79 |
|  | Warrior | 2012 |  | 62.67 | 22.80 | 36.4 | 1.289 | 19.2 | 44.2 | 14.9 | 6.5 | 0.7 | 1.5 | 6.9 | 90.5 | 7.6 | 1.9 | 4.004 | 347.48 | 74.57 | 2.71 |
|  |  | 2013 | 32.8 | 75.24 | 21.80 | 29.0 | 1.205 | 22.5 | 27.7 | 14.5 | 4.9 | 0.6 | 1.0 | 6.1 | 86.1 | 10.7 | 3.2 | 3.306 | 284.28 | 50.25 | 2.11 |
|  | Distillery | 2012 | 28.9 | 85.78 | 48.07 | 56.0 | 2.316 | 28.8 | 43.7 | 16.2 | 5.9 | 0.7 | 1.3 | 6.8 | 93.2 | 5.7 | 1.1 | 4.981 | 373.90 | 77.96 | 2.39 |
|  | Biofuel | 2012 | 96.6 | 90.22 | 34.50 | 38.2 | 2.304 | 28.6 | 38.5 | 15.2 | 8.1 | 1.1 | 2.0 | 8.7 | 91.3 | 6.9 | 1.8 | 3.952 | 349.59 | 48.51 | 2.03 |


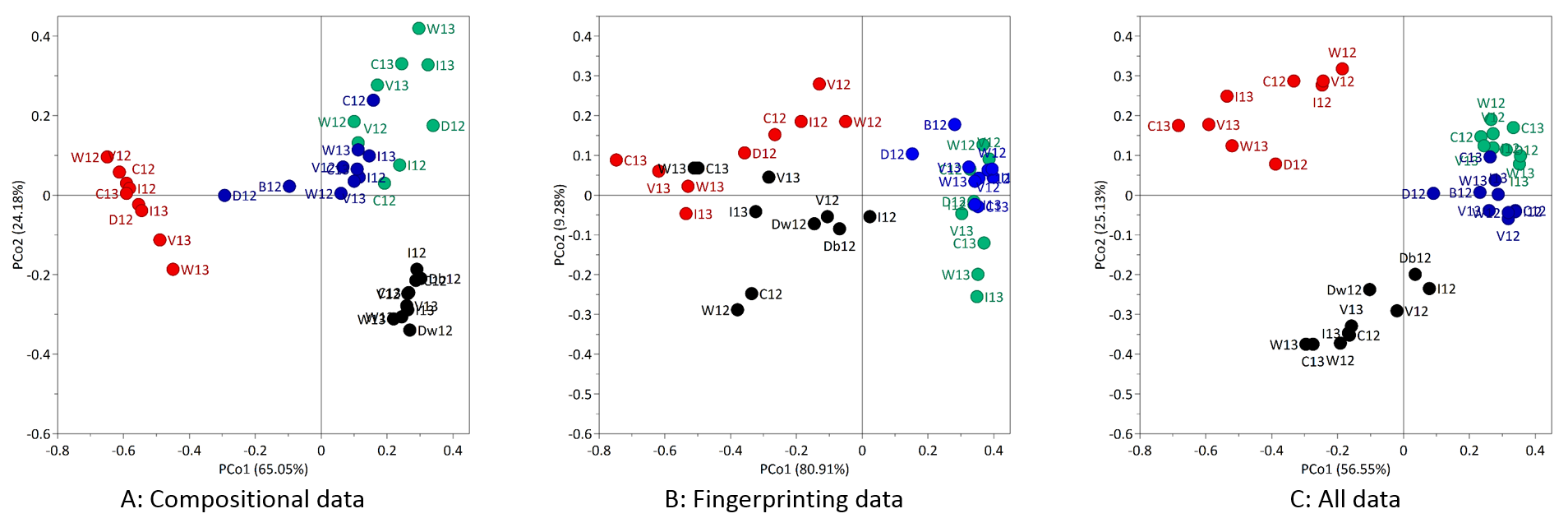


**Fig. S1.** Principal coordinates analysis (PCoA) plots for analyses of compositional data summarised in Table 1 (plot A), the fingerprinting data in Table 2 (plot B) and the combined data set Table S1 (plot C). These showed broadly similar separations although the degree of discrimination was clearly greater when the full dataset was used. The following coding of the samples is used, for cultivars: Claire (C), Istabraq (I), Viscount (V), Warrior (W), commercial distillery wheat (Dw) and barley (Db), and biofuel (B); followed by year: 2012 (12) and 2013 (13); and coloured by fraction type: wholemeal (WM, coloured black), thin stillage (TS, coloured red), wet distillers grains (WDG, coloured green) and distillers’ dried grains with solubles (DDGS, coloured blue). There is a clear separation of WM and TS from WDG and DDGS clusters in all 3 plots, and clear separation of TS from WM in plots A and C. This shows the similarity in nature of WDG and DDGS samples and the dissimilarity of TS to the other fractions. The percentage of the variation in the distances between the samples accounted for by each PCo is shown in brackets. In all cases, the first two PCos accounted for at least 81.68% of the variation in the distances between the samples and so only these two are retained for visualisation of the samples.


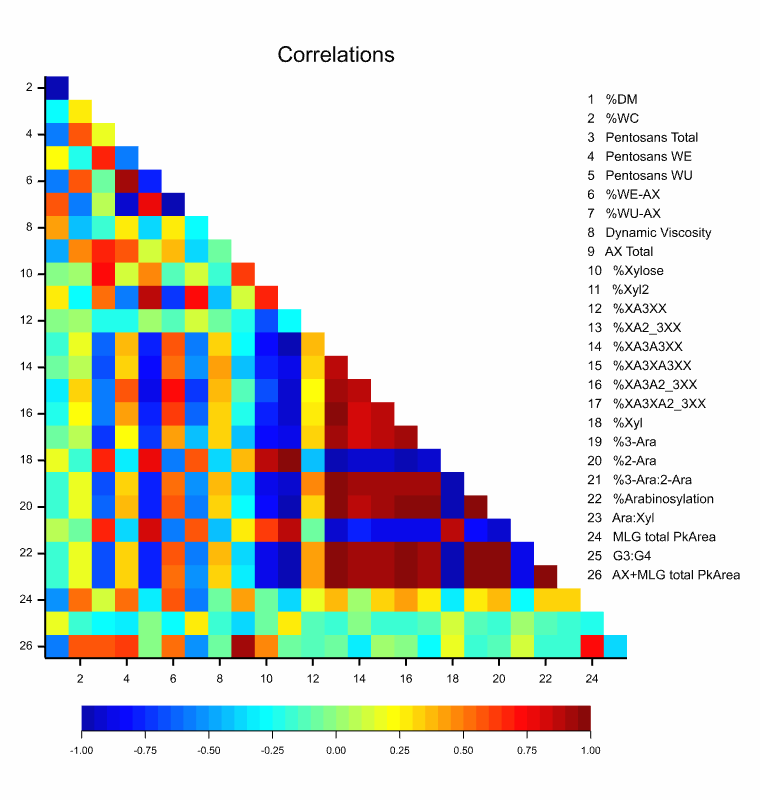


**Fig. S2.** Shade plot of the correlations between all variables. The key on the right indicates the number of the corresponding variable in the plot, and the scale bar below the plot shows the evaluation in colour of the range of correlations from -1 to +1.
